# The Prevalence and Burden of Psychiatric Disorders in Primary Health Care Visits in Asir Region Saudi Arabia

**DOI:** 10.7759/cureus.65253

**Published:** 2024-07-24

**Authors:** Asma Habbash, Yazeed S Alshahrani, Huda T Alshahrani, Salma S Alshahrani, Huda A Alshahrani, Reema S Alqahtani, Sara S Alqahtani, Ahmad Alqarni

**Affiliations:** 1 Family Medicine Department, King Khalid University, Abha, SAU; 2 Faculty of Medicine, King Khalid University, Abha, SAU; 3 Faculty of Pharmacy, King Khalid University, Abha, SAU

**Keywords:** asir region, kessler psychological distress scale, health visits, burden, prevalence, saudi arabia, primary care, psychological distress, mental health, psychiatric disorders

## Abstract

Background: Psychiatric disorders encompass a wide range of conditions that affect an individual's mental health and well-being. These disorders can manifest in various ways. The recognition, diagnosis, treatment, and referral of psychological conditions heavily rely on general practitioners, who handle consultations that involve a psychological aspect in at least one third of cases.

Objectives: To assess the prevalence and burden of common psychiatric disorders at primary health care centers (PHCs) using the Kessler Psychological Distress Scale (K6) in the general population, aged 18-65, and examine their symptom patterns and comorbidity.

Methodology: A descriptive online cross-sectional survey was conducted over a one-month period, spanning from September to October 2023. The survey targeted the population living in the Asir region. To fulfill the objectives, the K6 scale was used. Data analysis was carried out using the Statistical Package for the Social Sciences (SPSS) version 25. P values were considered statistically significant when they were ≤0.05.

Results: The survey included a total of 1,595 participants. Of these, 21.3% of respondents were male, while 78.7% were female. The majority of respondents fall within the 18-25 age group, accounting for 50.5% of the total. A significant portion of the population experiences some form of psychological distress, with 4.6% reporting low psychological distress, 36.1% reporting mild psychological distress, and a substantial 59.2% reporting severe psychological distress. Age (p-value=0.024), gender (p-value=0.001), educational level (p-value=0.001), occupation (p-value=0.008), and monthly income (p-value=0.001) had significant associations with the psychological distress score of psychiatric disorders.

Conclusion: The prevalence and burden of psychiatric disorders in primary health care visits in Saudi Arabia is a significant public health concern. Our findings showed that the majority of participants reported having severe to mild psychological distress.

## Introduction

Psychiatric disorders encompass a wide range of conditions that affect an individual's mental health and well-being. These disorders can manifest in various ways, including mood disturbances, anxiety, psychosis, and substance abuse [1]. Current estimates indicate that 50% of the population experience at least one mental disorder in their lifetime and that at least 25% have suffered a mental disorder in the past year [2]. Recognition, diagnosis, treatment, and referral depend overwhelmingly on general practitioners, at least one third of whose consultations have a direct and explicit psychological component [3]. 

In most health care systems, primary care doctors are the cornerstone of recognition, diagnosis, treatment, and specialist referral for all types of disorders, whether they are somatic, psychological, or both. The past two decades have witnessed a further emphasis of this role, particularly regarding the treatment of mental disorders in primary care. Several reasons account for this. First, mental disorders are extremely prevalent in the community, and much more than previously thought. Current epidemiological findings suggest that almost 50% of the population will experience at least one mental disorder in their lifetime, and at least 25% have suffered from a mental disorder during the past 12 months [4,5].

One of the challenges in addressing psychiatric disorders in primary care is the stigma and misconceptions surrounding mental health. Many individuals may be hesitant to seek help for their symptoms, and primary care providers may not always feel comfortable or confident in addressing mental health concerns. This can lead to under diagnosis and undertreatment of psychiatric disorders in primary care settings [2].

However, there is a growing recognition of the importance of integrating mental health care into primary care. Collaborative care models, which involve a team-based approach to managing psychiatric disorders, have been shown to be effective in improving patient outcomes. These models involve close collaboration between primary care providers, mental health professionals, and other support services to ensure comprehensive and coordinated care for patients with psychiatric disorders [1].

## Materials and methods

Study design and setting

A descriptive online cross-sectional survey was conducted over a 1-month period, spanning from September to October 2023. The survey targeted the population living in the Asir region.

Study population, sample size, and sampling technique

In this study, the sample size was determined using G power 3.1 with an assumed effect size of 0.1, an alpha error of 5%, and a power of 95%. The minimum sample size required to detect people with good knowledge was calculated to be 1,410. To account for a potential non-response rate of 10%, the sample size was increased to 1,595.

Inclusion Criteria

Participants were required to be male or female, at least 18 years old, living in the Asir region, and to have attended primary health care for any reason. The study population was recruited using a nonprobability sampling method (convenience and snowball sampling methods).

The questionnaire and data collection

To fulfill the objectives of our study, we used the Kessler Psychological Distress Scale (K6), which is a simple measure of psychological distress involving six questions about a person's emotional state.

Demographic Information

This section gathered data on participants' sex, age, education level, residence, smoking, and income. Each question was scored from 0 (none of the time) to 4 (all of the time). Scores for the six questions were then summed, yielding a minimum score of 0 and a maximum score of 24. Low scores indicate low levels of psychological distress, and high scores indicate high levels of psychological distress. In the COVID-19 public and health care workers survey, the K6 scores were divided as follows: a score between 0 and 4 indicated low/no psychological distress, a score between 5 and 12 indicated mild/moderate psychological distress, and a score between 13 and 24 indicated severe/serious psychological distress.

Statistical analysis

Responses from the participants were transcribed into an Excel spreadsheet. Data analysis was carried out using Statistical Package for the Social Sciences (SPSS) version 25. Descriptive statistics were conducted to summarize socio-demographic characteristics, knowledge scores, and the prevalence of psychiatric disorders. Inferential statistics, such as chi-square tests or logistic regression, were used to explore potential associations between socio-demographic variables and its levels. P values were considered statistically significant when they were ≤0.05. For questions where participants strongly agreed or agreed with a correct practice, the answers were considered accurate; conversely, if they strongly disagreed or disagreed with a particular practice, the answers were considered incorrect. Questions receiving correct answers from less than 90% of the participants were categorized as revealing a "Gap" in participant knowledge.

Ethical approval

Written informed consent was obtained from all participants before they completed the online questionnaire. All data collected was treated with strict privacy measures.

## Results

The study included a total of 1,595 participants. Regarding residency, data provided in Table 1 showed, 36.0% of respondents reported visiting health centers in the Asir region, while 64.0% did not. For those who visited health centers, the data further specifies the distribution among different centers in the region. The percentages vary for each center, with the highest percentage being 7.8% for the Classy center and the lowest being 2.6% for the Military center. Notably, 6.6% of respondents visited the Abha center, and 6.3% visited the Al Manhal Centre and Minsk Center each. In terms of age distribution, the data indicates that the majority of respondents fall within the 18-25 age group, accounting for 50.5% of the total. The 26-35 age group comprises 20.1% of respondents, followed by the 36-45 age group at 16.0% and the 45-65 age group at 13.4%. Gender-wise, the data shows that 21.3% of respondents are male, while 78.7% are female. This indicates a significant gender disparity within the sample. Education level is also well-documented, with 74.5% of respondents having a university education, 18.1% having completed high school, and 2.7% having attended middle school. Additionally, 4.7% fall under the "Other" category, signifying alternative educational backgrounds. Occupation distribution reveals that 41.1% of respondents are students, making it the most prominent occupational category within the sample. Other significant sectors include the educational sector at 15.7%, the "Other" category at 16.1%, and the industrial sector at 4.6%. The data also sheds light on the monthly income of respondents, with 56.6% earning less than 5000 Saudi Riyals, 28.1% earning more than 9000 Saudi Riyals, and 15.4% falling within the 5000-9000 Saudi Riyals income bracket.

**Table 1:** Socio-demographic characteristics of participants (n=1,595)

Parameter		No.	%
Did you go to one of the health centers in the Asir region?	Yes	574	36.0
	No	1,021	64.0
If your answer is (yes), mention the center , If your answer is (no), write none	Abha	38	6.6
	One of Rafidah	36	6.3
	Classy	45	7.8
	Saudi German	35	6.1
	Industrial	37	6.4
	Military	15	2.6
	Diabetes center	25	4.4
	Care center	39	6.8
	Northern Seed Healthcare Center	18	3.1
	Al-Harida Center	21	3.7
	New neighborhood center	29	5.1
	Al-Rabea neighborhood center	19	3.3
	Al Manhal Centre	36	6.3
	Minsk Center	39	6.8
	Al-Qabil Center	25	4.4
	Al-Azizia Health Center	35	6.1
	Majarda	24	4.2
	Durrat Al Minsk Center	28	4.9
	Al-Dhabat District Health Center	25	4.4
Age	18-25	806	50.5
	26-35	320	20.1
	36-45	255	16.0
	45-65	214	13.4
Gender	Male	339	21.3
	Female	1,256	78.7
Education Level	Middle school	43	2.7
	High school	288	18.1
	University	1,189	74.5
	Other	75	4.7
Occupation	Educational sector	251	15.7
	Industrial sector	6	.4
	Military sector	73	4.6
	Private sector	71	4.5
	Student	655	41.1
	The sector is healthy	159	10.0
	Government sector	2	.1
	Housewife	70	4.4
	Unemployed	42	2.6
	Retired	10	.6
	Other	256	16.1
Monthly Income (in Saudi Riyals)	Less than 5,000	902	56.6
	5,000-9,000	245	15.4
	More than 9,000	448	28.1

Table 2 showed that for the feeling of nervousness, the data indicates that 14.9% of respondents experienced this emotion "ALL" of the time, while 28.5% felt nervous "MOST" of the time. Additionally, 39.6% felt nervous "SOME" of the time, 13.0% felt nervous "A LITTLE" of the time, and 4.0% reported feeling "NONE" of the time. Regarding feelings of hopelessness, 9.2% of participants reported feeling this way "ALL" of the time, with 20.2% feeling hopeless "MOST" of the time. Furthermore, 39.2% felt hopeless "SOME" of the time, 21.6% felt hopeless "A LITTLE" of the time, and 9.8% did not experience feelings of hopelessness at all. The data also reveals that 11.5% of individuals felt restless or fidgety "ALL" of the time, while 21.5% experienced this feeling "MOST" of the time. Moreover, 34.5% felt restless or fidgety "SOME" of the time, 19.2% felt this way "A LITTLE" of the time, and 13.2% did not experience these sensations. For feelings of profound sadness, 14.0% of respondents felt this way "ALL" of the time, with 24.3% feeling so sad "MOST" of the time. Additionally, 32.7% experienced this emotion "SOME" of the time, 17.4% felt it "A LITTLE" of the time, and 11.6% did not feel profoundly sad at all. In terms of perceiving everything as an effort, 20.9% of participants felt this way "ALL" of the time, while 27.4% felt this way "MOST" of the time. Furthermore, 33.5% experienced this feeling "SOME" of the time, 11.0% felt it "A LITTLE" of the time, and 7.2% did not feel that everything was an effort. Lastly, regarding feelings of worthlessness, 13.4% of individuals felt this way "ALL" of the time, with 16.0% feeling worthless "MOST" of the time. Additionally, 29.3% experienced this emotion "SOME" of the time, 18.2% felt it "A LITTLE" of the time, and 23.0% did not feel worthless at all.

**Table 2:** Burden of psychiatric disorders in primary health care visits (n=1,595)

Parameter	ALL	MOST	SOME	A LITTLE	NONE
How often did you feel nervous	237 14.9%	454 28.5%	632 39.6%	208 13.0%	64 4.0%
How often did you feel hopeless	147 9.2%	322 20.2%	625 39.2%	345 21.6%	156 9.8%
How often did you feel restless or fidget	183 11.5%	343 21.5%	551 34.5%	307 19.2%	211 13.2%
How often did you feel so sad that nothing could cheer you	223 14.0%	387 24.3%	522 32.7%	278 17.4%	185 11.6%
How often did you did you feel that everything was an effort	333 20.9%	437 27.4%	535 33.5%	175 11.0%	115 7.2%
How often did you feel worthless	214 13.4%	255 16.0%	468 29.3%	291 18.2%	367 23.0%

**Figure 1:**
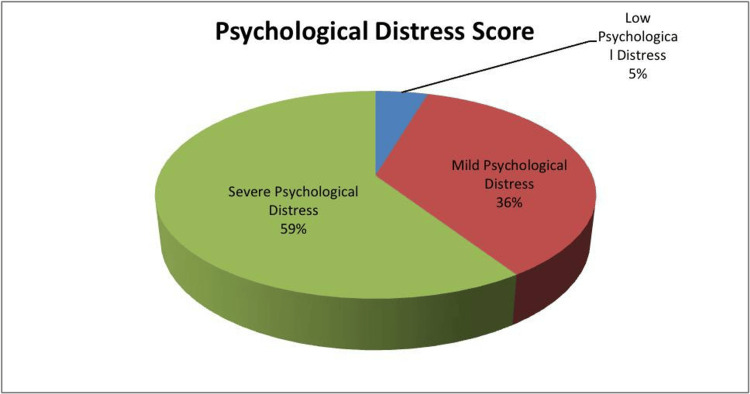
Psychological distress score of psychiatric disorders in primary health care visits

It is evident from Figure 1 that a significant portion of the population experiences some form of psychological distress, with 4.6% reporting low psychological distress, 36.1% reporting mild psychological distress, and a substantial 59.2% reporting severe psychological distress.

Table 3 indicates that among those who visited health centers in the Asir region, 20.6% reported severe psychological distress, 13.6% reported mild psychological distress, and 1.8% reported low psychological distress. The p-value for this parameter is 0.500, suggesting no significant association between visiting health centers in the Asir region and psychological distress.

**Table 3:** Association between socio-demographic characteristics and psychological distress score of psychiatric disorders in primary health care visits (n=1,595)

Parameter		Psychological distress score			Total (n=1,595)	P value
		Severe Psychological Distress	Mild Psychological Distress	Low Psychological Distress		
Did you go to one of the health centers in the Asir region?	Yes	329	217	28	574	0.500
		20.6%	13.6%	1.8%	36.0%	
	No	616	359	46	1,021	
		38.6%	22.5%	2.9%	64.0%	
Age	18-25	541	238	27	806	0.001
		33.9%	14.9%	1.7%	50.5%	
	26-35	188	120	12	320	
		11.8%	7.5%	0.8%	20.1%	
	36-45	133	105	17	255	
		8.3%	6.6%	1.1%	16.0%	
	45-65	83	113	18	214	
		5.2%	7.1%	1.1%	13.4%	
Gender	Male	168	138	33	339	0.001
		10.5%	8.7%	2.1%	21.3%	
	Female	777	438	41	1,256	
		48.7%	27.5%	2.6%	78.7%	
Education Level	Middle school	23	14	6	43	0.008
		1.4%	0.9%	0.4%	2.7%	
	High school	178	96	14	288	
		11.2%	6.0%	0.9%	18.1%	
	University	704	439	46	1,189	
		44.1%	27.5%	2.9%	74.5%	
	Other	40	27	8	75	
		2.5%	1.7%	0.5%	4.7%	
Occupation	Educational sector	115	120	16	251	0.001
		7.2%	7.5%	1.0%	15.7%	
	Industrial sector	5	1	0	6	
		0.3%	0.1%	0.0%	0.4%	
	Military sector	38	29	6	73	
		2.4%	1.8%	0.4%	4.6%	
	private sector	37	31	3	71	
		2.3%	1.9%	0.2%	4.5%	
	student	440	194	21	655	
		27.6%	12.2%	1.3%	41.1%	
	The sector is healthy	104	51	4	159	
		6.5%	3.2%	0.3%	10.0%	
	Governmental sector	1	0	1	2	
		0.1%	0.0%	0.1%	0.1%	
	Housewife	34	32	4	70	
		2.1%	2.0%	0.3%	4.4%	
	Unemployed	29	11	2	42	
		1.8%	0.7%	0.1%	2.6%	
	Retired	2	7	1	10	
		0.1%	0.4%	0.1%	0.6%	
	Other	140	100	16	256	
		8.8%	6.3%	1.0%	16.1%	
Monthly Income (in Saudi Riyals)	Less than 5,000	587	285	30	902	0.001
		36.8%	17.9%	1.9%	56.6%	
	5,000 - 9,000	139	92	14	245	
		8.7%	5.8%	0.9%	15.4%	
	Over 9,000	219	199	30	448	
		13.7%	12.5%	1.9%	28.1%	

Age

The table reveals a statistically significant association between age and psychological distress, with p-values ranging from 0.001 to 0.001 across different age groups. The percentage of respondents reporting severe psychological distress decreases as age increases, with the highest percentage (33.9%) in the 18-25 age group and the lowest percentage (5.2%) in the 45-65 age group.

Gender

The data indicates a statistically significant association between gender and psychological distress, with a p-value of 0.001. Female respondents reported higher percentages of severe psychological distress (48.7%) compared to male respondents (10.5%).

Education level

There is a statistically significant association between education level and psychological distress, with a p-value of 0.008. Respondents with a university education reported the highest percentage of severe psychological distress (44.1%), followed by high school (11.2%) and middle school (1.4%).

Occupation

The table shows a statistically significant association between occupation and psychological distress, with p-values ranging from 0.001 to 0.001 across different occupational sectors. Students reported the highest percentage of severe psychological distress (27.6%), followed by the educational sector (7.2%) and the "other" category (8.8%).

Monthly income

There is a statistically significant association between monthly income and psychological distress, with p-values ranging from 0.001 to 0.001 across different income brackets. Respondents with a monthly income of less than 5,000 Saudi Riyals reported the highest percentage of severe psychological distress (36.8%), followed by those with an income of 5,000-9,000 Riyals (8.7%) and over 9,000 Riyals (13.7%).

## Discussion

The prevalence and burden of psychiatric disorders in primary health care visits in Saudi Arabia is a topic of great importance and significance. Mental health issues are a growing concern worldwide, and it is crucial to understand the extent of these issues in order to provide appropriate support and resources to those in need. In Saudi Arabia, like many other countries, mental health disorders are often stigmatized and individuals may be hesitant to seek help. This can lead to underreporting and under diagnosis of psychiatric disorders, making it difficult to accurately assess the prevalence and burden of these conditions. However, recent studies have shed light on the significant impact of psychiatric disorders on primary health care visits in Saudi Arabia [1-3].

The burden of psychiatric disorders in primary health care visits in Saudi Arabia extends beyond the individual level, impacting families, communities, and the overall healthcare system. Individuals with untreated mental health disorders may experience decreased quality of life, impaired functioning, and increased risk of chronic physical health conditions. Additionally, the economic burden of untreated mental health disorders can be substantial, as it may lead to increased healthcare utilization, productivity loss, and disability [4].

Our study showed that most of our participants had psychological distress, as 59.2% reported suffering severe psychological distress score, and 36.1% reporting having mild psychological distress score. Similarly, a study conducted in the capital of Saudi Arabia in 2018 showed that around 28.5% of the patients exhibited mental problems [5]. This is highly similar to two studies that discovered a prevalence of mental disorders, such as depression, anxiety, somatization, or panic disorders, among 431 outpatients using the Patient Health Questionnaire (PHQ). The prevalence of minor mental illness among 609 outpatients in primary care settings, as measured by the Rahim Anxiety-Depression Scale, was found to be 33.4% and 30.5%, respectively [6,7]. Another study in Qatar showed that primary care clinics in Qatar were found to have a rather high frequency of current psychiatric illnesses. The most prevalent problems were major depression disorders, any anxiety disorder, any impulse control condition, and mood disorder, affecting approximately 20% of patients each [8].

Our study showed that residency in Asir, age, gender, educational level, occupation, and monthly income had significant association with psychological distress score of psychiatric disorders, with p-values 0.024, 0.001, 0.001, 0.008, 0.001, and 0.001 respectively. As our results found that residents in Asir tend to have higher psychological distress, participants in age group 18-25 tend to have higher psychological distress, females have more severe psychological distress, university degree holders have more severe psychological distress than others, students have more severe psychological distress than other groups, and finally participants who have monthly income less than 5,000 Riyals have more severe psychological distress than participants with higher income. Similarly another study showed that the prevalence of anxiety disorders in women was markedly higher than in males, with rates of 21.4% compared to 12.1% [5].

Similarly, the prevalence of major depression disorders in women was 22.0%, greatly above the rate of 13.8% observed in men. Prince et al. observed that women have a greater susceptibility to common mental diseases, with a female to male sex ratio of 1.5:1. Societally, the position of women in this culture is undergoing transformation [1]. However a recent study has found that the decline in the occurrence of mental depression episodes among the elderly is significantly greater in industrialized countries compared to developing ones [9].

Addressing the prevalence and burden of psychiatric disorders in primary health care visits in Saudi Arabia requires a multifaceted approach. This includes increasing awareness and reducing stigma surrounding mental health, integrating mental health services into primary care settings, and providing training and support for healthcare professionals to effectively identify and manage psychiatric disorders. Additionally, efforts to promote mental health literacy and provide accessible and culturally sensitive mental health services are essential in addressing the needs of individuals with psychiatric disorders in Saudi Arabia.

It is also important to note some limitations of the study. First, the study may not capture the full extent of psychiatric disorders in the region, as it relies on data from primary health care visits, potentially excluding individuals who do not seek medical attention for their mental health concerns. Additionally, the study may be limited by the accuracy and completeness of the diagnostic information recorded in the health care records. Furthermore, the study's findings may not be generalizable to other regions or populations.

Despite these limitations, the study contributes to our understanding of psychiatric disorders in primary health care settings in the Asir Region. The findings of this study can have important future implications for healthcare policy and resource allocation in Saudi Arabia. By understanding the prevalence and burden of psychiatric disorders in primary care settings, healthcare providers and policymakers can better tailor mental health services to meet the needs of the population. Additionally, this study can also serve as a foundation for further research and interventions aimed at improving mental health outcomes in the Asir Region and beyond. Overall, the results of this study have the potential to inform and shape the future of mental health care in Saudi Arabia.

## Conclusions

In conclusion, the prevalence and burden of psychiatric disorders in primary health care visits in Saudi Arabia represent a significant public health concern. Our findings indicate that a substantial majority of participants experience psychological distress, with 59.2% reporting severe distress and 36.1% reporting mild distress. These high rates of psychological distress highlight the urgent need for comprehensive mental health services within primary care settings.

The study reveals significant associations between psychological distress and various socio-demographic factors, including age, gender, educational level, occupation, and income. Younger individuals, particularly those aged 18-25, females, university-degree holders, students, and those with lower income levels are more likely to experience severe psychological distress. These insights underscore the necessity for targeted interventions that consider these demographic variables to effectively address mental health disparities.

Moreover, the integration of mental health services into primary care is crucial. Collaborative care models that involve a multidisciplinary approach, combining the efforts of primary care providers, mental health professionals, and support services, have proven effective in improving patient outcomes. These models should be adopted and expanded within the Saudi Arabian healthcare system to ensure that individuals with psychiatric disorders receive timely and appropriate care.

Addressing the stigma associated with mental health is equally important. Public health campaigns and educational programs aimed at increasing mental health literacy and reducing stigma can encourage individuals to seek help and support. Additionally, training primary care providers to recognize and manage psychiatric disorders can enhance the overall quality of care.

The findings of this study can inform healthcare policy and resource allocation in Saudi Arabia. By understanding the prevalence and burden of psychiatric disorders, policymakers can better allocate resources and design interventions that address the specific needs of the population. Future research should continue to explore the factors contributing to mental health issues and evaluate the effectiveness of implemented interventions.

Overall, the study highlights the critical need for a comprehensive approach to mental health care in primary health care settings. By addressing the identified gaps and implementing targeted strategies, we can improve mental health outcomes and reduce the burden of psychiatric disorders on individuals, families, and the healthcare system as a whole.

## Appendices

The Kessler Psychological Distress Scale (K6) questionnaire consists of the following questions, each rated on a scale from 0 (none of the time) to 4 (all of the time):

1. During the past 30 days, about how often did you feel nervous? 2. During the past 30 days, about how often did you feel hopeless? 3. During the past 30 days, about how often did you feel restless or fidgety? 4. During the past 30 days, about how often did you feel so sad that nothing could cheer you up? 5. During the past 30 days, about how often did you feel that everything was an effort? 6. During the past 30 days, about how often did you feel worthless?

## References

[1] Prince M, Patel V, Saxena S, Maj M, Maselko J, Phillips MR, Rahman A (2007). No health without mental health. Lancet.

[2] Bener A, Ghuloum S (2011). Gender differences in the knowledge, attitude and practice towards mental health illness in a rapidly developing Arab society. Int J Soc Psychiatry.

[3] Ghuloum S, Bener A, Dafeeah EE, Zakareia AE, El-Amin A, El-Yazidi T (2014). Prevalence of common mental disorders in general practice attendees: Using World Health Organization Composite International Diagnostic Interview (WHO-CIDI) in Qatar. Int J Clin Psychiatry Ment Health.

[4] Bener A, Dafeeah EE, Chaturvedi SK, Bhugra D (2013). Somatic symptoms in primary care and psychological comorbidities in Qatar: neglected burden of disease. Int Rev Psychiatry.

[5] Alghadeer SM, Alhossan AM, Al-Arifi MN, Alrabiah ZS, Ali SW, Babelghaith SD, Altamimi MA (2018). Prevalence of mental disorders among patients attending primary health care centers in the capital of Saudi Arabia. Neurosciences (Riyadh).

[6] Becker S, Al Zaid K, Al Faris E (2002). Screening for somatization and depression in Saudi Arabia: a validation study of the PHQ in primary care. Int J Psychiatry Med.

[7] Al-Khathami AD, Ogbeide DO (2002). Prevalence of mental illness among Saudi adult primary-care patients in Central Saudi Arabia. Saudi Med J.

[8] Bener A, Abou-Saleh MT, Dafeeah EE, Bhugra D (2015). The prevalence and burden of psychiatric disorders in primary health care visits in qatar: too little time?. J Family Med Prim Care.

[9] Kessler RC, Birnbaum HG, Shahly V (2010). Age differences in the prevalence and co-morbidity of DSM-IV major depressive episodes: results from the WHO World Mental Health Survey Initiative. Depress Anxiety.

